# Estimating true standard deviations

**DOI:** 10.3389/fpsyg.2014.00235

**Published:** 2014-03-18

**Authors:** David Trafimow

**Affiliations:** Department of Psychology, New Mexico State UniversityLas Cruces, NM, USA

**Keywords:** standard deviation, classical test theory, classical true score theory, true standard deviations, random measurement error

A basic statistic that students learn about in their classes is the standard deviation. Like any statistic, standard deviations are influenced by systematic factors and randomness. I propose that researchers should report “corrected” or “true” standard deviations and I show how to calculate them.

The notion of “true” statistics comes out of *classical test theory* (see Lord and Novick, 1968; Gulliksen, 1987 for reviews). This theory commences with the definition of a “true score”—as the expectation across an infinite set of independent responses—and with an assumption that an observed score equals the true score plus error (*X* = *T* + *E*). Thus, measures of constructs necessarily include random variance, as well as non-random variance. Many statistics—possibly the most famous of which is the correlation coefficient—are influenced by random measurement error (e.g., Spearman, 1904). The deleterious effects of random measurement error are well known, and many statistical packages contain provisions for correcting correlation coefficients, so these corrected correlations can be used in complex path analyses and structural equation analyses, thereby increasing their accuracy (Skrondal and Rabe-Hesketh, 2004). In addition, Baguley (2009) has addressed the correction of effect sizes.

Given that it is widely accepted that “corrected” or “true” statistics, uncontaminated by random measurement error, are necessary for complex analyses such as those mentioned above, why not obtain them even for simple cases such as standard deviations? According to the classical theory, the reliability of a measure (ρ_*XX*′_) equals the ratio of the true score variance (symbolized as σ^2^_*T*_) to observed score variance (symbolized as σ^2^_*X*_) or $\rho_{XX^{\prime }}=\frac{\sigma_{T}^{2}}{\sigma_{X}^{2}}$. By rearranging the terms, it is possible to isolate the true score variance—that is, the variance of the measure with random measurement error removed, as is shown in Equation 1 below.

(1) $$\sigma_{T}^{2}=\rho_{XX^{\prime }}\sigma_{X}^{2}$$

Because researchers usually do not have access to population parameters, it is necessary to estimate them from data. The estimated true score variance (*est* σ^2^_*T*_) can be obtained from Equation 2 if the researcher has collected the requisite data to obtain the reliability of the measure of the construct (*r*_*XX*′_) and its variance in the experiment (*s*^2^_*X*_).

(2) $$est\;\sigma_{T}^{2}=r_{XX^{\prime }}s_{X}^{2}$$

Consider a researcher who performs an experiment to determine whether participants who receive an intervention have more favorable attitudes toward recycling than participants in a no intervention control condition. The reliability of the attitude measure is 0.7, and the variance in the experimental and control group is 0.95 and 1.3, respectively. Understanding that the observed variances are contaminated by random measurement error, the researcher wishes to estimate the variance of the attitude measure in the two conditions uncontaminated by random measurement error. In the intervention condition, the estimated attitude variance is as follows: *est* σ^2^_*T*_ = (0.7) (0.95) = 0.665. In the control condition, it is as follows: *est* σ^2^_*T*_ = (0.7) (1.3) = 0.91. Note that the true attitude variance in the intervention condition, uncontaminated by random measurement error, differs substantially from the observed variance of the attitude measure contaminated by random measurement error (0.665 vs. 0.95) and this also is so in the control condition (0.91 vs. 1.3). Standard deviations can be obtained, as usual, with square roots of variances. In the example, these *true* standard deviations would be 0.815 and 0.954 for the intervention group and control group, respectively, in contrast to the *observed* values of 0.975 and 1.140. The observed standard deviations actually overestimate the true standard deviations. Given the ease with which it is possible to obtain estimates of true standard deviations, provided that the reliability of the measure is known, it makes sense to report true standard deviations either in place of, or in addition to, observed standard deviations.

There is, however, a complication with the foregoing. Specifically, reliability coefficients are not perfectly precise (e.g., Zimmerman, 2007) and that imprecision might be carried over into the computed true standard deviation. One way of handling this is to define an interval (Hayduk, 1987) but a caveat is important here. As an example, when a researcher defines a 95% confidence interval, this does not imply that the parameter of interest has a 95% chance of being in the interval. Rather, it means that if there were an infinite number of replications, and a confidence interval were computed each time, 95% of the computed intervals would enclose the parameter. Unfortunately, there is no known way to make the valid inverse inference about the probability that a given interval encloses the parameter of interest. Nevertheless, intervals can be useful though defining them is necessarily somewhat arbitrary. Hunter and Schmidt (2004) discussed issues pertaining to confidence intervals for true statistics.

Suppose, that a dependent measure has been tested—either in the literature or in pilot research—and the reliability equals 0.7. We might define a reliability interval as within 10 points in either direction (0.6–0.8) and use Equation 2 to obtain the standard deviations based on the endpoints of the interval. If we imagine that the observed variance is 2 (*SD* = 1.41), the interval for that variance would range from 1.20 to 1.60, and so interval for the corresponding standard deviation would range from 1.09 to 1.26.

Alternatively, as for many personality tests, several reliability coefficients may be reported, such as between 0.7 and 0.8. It might be reasonable to go ahead and define an interval empirically (0.7–0.8), based on the literature. Or if there have been many reports, one could define an empirical interval based on a range such as the middle two quartiles of reported values. However, the interval is defined, the procedure for using it would be similar to that illustrated earlier.

My argument can be summarized easily. Researchers agree that standard deviations matter. But should researchers report standard deviations that are contaminated by random measurement error, uncontaminated by random measurement error, or both. I submit that there will be times when it is desirable to know standard deviations that are uncontaminated by random measurement error, and Equation 2 provides a way by which they can be attained easily. Therefore, I advocate reporting true standard deviations in addition to observed standard deviations.
